# Transcriptional profiling of predator-induced phenotypic plasticity in *Daphnia pulex*

**DOI:** 10.1186/s12983-015-0109-x

**Published:** 2015-07-25

**Authors:** Andrey Rozenberg, Mrutyunjaya Parida, Florian Leese, Linda C. Weiss, Ralph Tollrian, J. Robert Manak

**Affiliations:** Department of Animal Ecology, Evolution and Biodiversity, Ruhr University Bochum, Universitaetsstrasse 150, Bochum, 44801 Germany; Departments of Biology and Pediatrics and the Roy J. Carver Center for Genomics, 459 Biology Building, University of Iowa, Iowa City, IA 52242 USA; Environmental Genomics Group, School of Biosciences, University of Birmingham, Birmingham, B15 2TT UK; Present address: University of Duisburg-Essen, Aquatic Ecosystems Research, Universitaetsstrasse 5, Essen, 45141 Germany

**Keywords:** Daphnia, Inducible defences, Phenotypic plasticity, Predator-prey interaction, RNA-Seq, Transcriptomics, Morphology, Gene-environment interactions

## Abstract

**Background:**

Predator-induced defences are a prominent example of phenotypic plasticity found from single-celled organisms to vertebrates. The water flea *Daphnia pulex* is a very convenient ecological genomic model for studying predator-induced defences as it exhibits substantial morphological changes under predation risk. Most importantly, however, genetically identical clones can be transcriptionally profiled under both control and predation risk conditions and be compared due to the availability of the sequenced reference genome. Earlier gene expression analyses of candidate genes as well as a tiled genomic microarray expression experiment have provided insights into some genes involved in predator-induced phenotypic plasticity. Here we performed the first RNA-Seq analysis to identify genes that were differentially expressed in defended vs. undefended *D. pulex* specimens in order to explore the genetic mechanisms underlying predator-induced defences at a qualitatively novel level.

**Results:**

We report 230 differentially expressed genes (158 up- and 72 down-regulated) identified in at least two of three different assembly approaches. Several of the differentially regulated genes belong to families of paralogous genes. The most prominent classes amongst the up-regulated genes include cuticle genes, zinc-metalloproteinases and vitellogenin genes. Furthermore, several genes from this group code for proteins recruited in chromatin-reorganization or regulation of the cell cycle (cyclins). Down-regulated gene classes include C-type lectins, proteins involved in lipogenesis, and other families, some of which encode proteins with no known molecular function.

**Conclusions:**

The RNA-Seq transcriptome data presented in this study provide important insights into gene regulatory patterns underlying predator-induced defences. In particular, we characterized different effector genes and gene families found to be regulated in *Daphnia* in response to the presence of an invertebrate predator. These effector genes are mostly in agreement with expectations based on observed phenotypic changes including morphological alterations, i.e., expression of proteins involved in formation of protective structures and in cuticle strengthening, as well as proteins required for resource re-allocation. Our findings identify key genetic pathways associated with anti-predator defences.

**Electronic supplementary material:**

The online version of this article (doi:10.1186/s12983-015-0109-x) contains supplementary material, which is available to authorized users.

## Background

The common freshwater micro-crustacean *Daphnia* has become a model organism for many biological disciplines [1–6]. The extensive knowledge of its ecology [5, 7, 8] and its biological responses to environmental changes [3, 9, 10] together with the availability of genomic resources [4] make the system highly attractive for evolutionary ecology research and provides the unique opportunity to study ecological traits with the aid of emerging molecular biological tools. One of the most intriguing ecological responses of *Daphnia* species to environmental changes is their ability to develop different phenotypes given the same genetic background, a phenomenon called phenotypic plasticity. Prominent examples of phenotypic plasticity include inducible defences.

Inducible defences are interpreted as adaptations to heterogeneous predation risks and are found in many organisms from protists to vertebrates [11–13]. *Daphnia* evolved sensitivity against specific chemical compounds, which are unintentionally emitted by their predators. These so-called kairomones serve as signals which prompt the daphnid prey to develop individuals which are better defended. Previous work has shown that different predators, e.g. fish and the phantom midge *Chaoborus* spp., can induce different, sometimes opposite phenotypic reactions in the same species or clone [10, 14–17]. This means that the genome must encode multiple developmental programs triggered by environmental conditions. Induced defences in *Daphnia* include prominent morphological modifications: from tiny cuticular teeth to very elongated tail and head spines, helmets or even giant crests [18–21], but also changes in life history and different behaviours, which ultimately all act as deterrents to encounter, capture and ingestion by the predator [9, 22–25].

In the model species *Daphnia pulex*, kairomones from the phantom-midge larvae *Chaoborus* trigger production of neck-teeth, the most easily detectable trait, and overall hardening of the cuticle [26]. These external, cuticle-associated alterations effectively protect juveniles from predation [27, 28]. At the same time, induced *D. pulex* females shift resources from reproduction to somatic growth, thereby reaching maturity at a larger size and producing less but larger offspring [29–31]. Vertical migration is deemed to comprise the main behavioural reaction to the presence of the predator in *D. pulex*: thus, *Chaoborus*-induced specimens prefer shallower depths in comparison to control specimens [32, 33]. *Chaoborus* is an ambush predator, such that *Daphnia* is also expected to reduce its swimming speed, although in the case of *D. pulex* this habit is displayed only by some clones [34–36] (LCW, unpublished observations).

Instability of environmental conditions (periodicity of predation risk, different predators) and costs of defences explain the inducible nature of the defensive morphs. This is also in line with the fact that the neck-teeth are present only in certain juvenile instars when the daphnids reach preferred prey size of their gape-limited predators [10, 13, 29, 37, 38].

Based on the experimental evidence we can make the following predictions regarding the underlying functional classes of effector genes that might contribute to *Daphnia*’s anti-predatory response (Fig. 1):

1. the structural changes in the cuticle are expected to mirror changes in the amounts/types of cuticle-associated proteins;

**Fig. 1:**
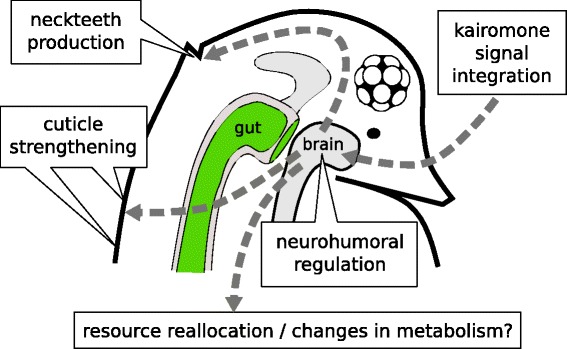
Presumptive scheme of physiological and morphological changes during kairomone-induction in *D. pulex*
2. life history modifications are expected to be controlled by several physiological changes affecting both somatic growth and reproduction;
3. one can predict that other metabolic functions should be down regulated under predation risk in order to allocate energy resources primarily to the above mentioned pathways;
4. all levels of the response must ultimately be controlled by cascades of receptors, humoral factors, signalling pathways and transcription factors.

Currently, the technique of choice suitable for addressing response patterns in gene expression at a genome-wide level with potentially unlimited depth of coverage is RNA-Seq [39–41]. The availability of the *D. pulex* draft genome [4] greatly facilitates the power of such analyses in that RNA-Seq reads can be specifically mapped to a particular genomic location. Investigations of the genome-environment interactions in *Daphnia* are ongoing, with the results of the first analyses of differential gene expression patterns in ecological experiments recently becoming available [42–45]. A number of features have been discovered that point to an ecological responsiveness of the *D. pulex* genome; e.g. a large overall number of genes, organized in the many families of paralogous genes that in many cases do not show homologies to genes in other organisms, but show differential expression under different environmental conditions [4, 46–49].

While preliminary analysis of the transcriptomic responses of *D. pulex* to the predator was performed earlier [4], gene expression was assessed with tiling microarrays and was restricted to the second juvenile instar, after the onset of neck-teeth production. Here we aimed at providing the first whole-genome analysis of gene expression changes involved in formation of predator-induced defences in *D. pulex*. To accomplish this, we apply the most versatile technique to study transcriptomes, RNA-Seq and focus on the first juvenile instar, the developmental point at which the defence is expected to unfold.

## Material and methods

### *Daphnia* clone used in the experiment

In contrast to the clone chosen for genome sequencing by the Daphnia Genomics Consortium (“TCO”), the clone we utilized for the experiment (designated as “R9”) is known to show pronounced production of defensive morphs in the presence of the phantom midge larvae *Chaoborus* [4]. It originates from Canada and according to mitochondrial markers belongs to the so-called Panarctic *Daphnia pulex* clade. The TCO clone in turn belongs to a group of populations united under the name “*Daphnia arenaria*” which is likely of hybrid origin with its mitochondrial genome coming from the same clade as R9, while nuclear markers point to closer relationships with North American *Daphnia pulicaria* [4, 50, 51].

### Experiment

We utilized a simple experimental design: one pooled series of *Daphnia pulex* juveniles exposed to *Chaoborus* and a control set of specimens without predator induction. Fifty age-synchronized specimens of the *D. pulex* R9 clone, served as the founding generation for the experimental animals. For induction, the mothers bearing late embryos were exposed to the *Chaoborus* larvae contained in a net cage and fed with 100 juvenile daphnids. Progeny obtained from the induced and control mother specimens was collected at the first juvenile instar, and stored in RNA Later (Qiagen, Hilden, Germany) for 24 h. Subsequently, specimens were stored at -20 °C until RNA extraction. Three independent induction experiments were performed leading to 90 juveniles in each group (induction and control) in total, which were pooled together to level individual variation.

### RNA extraction and sequencing

Total RNA was extracted with the TRIzol Reagent (Life Technologies, Carlsbad, CA, USA) according to the manufacturer’s protocol with modifications. DNA was further depleted with DNase treatment (TurboDNase, Life Technologies, Carlsbad, CA, USA) and its absence was confirmed afterwards via PCR with primers spanning exon-intron boundaries in an *α*-tubulin gene. Quality and amount of the purified RNA in the samples was analyzed on the Experion System (Bio-Rad, Hercules, CA, USA) with the aid of the Experion RNA StdSens Analysis Kit according to the supplied manual. The samples were shipped to Otogenetics (Norcross, GA, USA) for library preparation and sequencing. cDNA was synthesized with random hexamers after rRNA depletion. Size-selected cDNA fragments (250–300 bp range) were sequenced on an Illumina HiSeq 2000 from both ends. Overall, two sequencing runs were performed yielding 10–20 million 100 bp read pairs per sample.

Quality of the reads was analyzed with FastQC v. 0.10.1 (http://www.bioinformatics.babraham.ac.uk/projects/fastqc) and necessary filtering steps were performed with trimmomatic v. 0.22 (http://www.usadellab.org/cms/index.php?page=trimmomatic). Since a considerable contamination of the data with adapter sequences and non-coding RNAs (primarily, rRNA) was detected in the first experiment, we performed two rounds of trimmomatic treatment: removal of reads showing similarity to sequences of non-coding RNA (100 bp tiling fragments with 50 bp overlaps were used as queries) (ILLUMINACLIP:non_coding_rnas:4:40:12 MINLEN:81, for positive matches the whole pair was discarded) and adapter and quality trimming on the second step (ILLUMINACLIP:adapters:2:40:9 LEADING:15TRAILING:15MINLEN:36). After each step of contamination removal a quality control analysis using FastQC was performed to check the validity of our data processing steps.

The raw reads are available from the NCBI Sequence Read Archive (BioProject accession number: PRJNA287609).

### Assembly

We employed two principal alternative approaches to assemble the transcriptome: mapping-oriented and *de novo* assembly. For mapping we took the latest set of scaffolds for the *Daphnia pulex* genome: the 06.09.2005 version with further filtering steps as available on http://genome.jgi-psf.org/Dappu1 (5,191 scaffolds with the total length of 197,261,574 bp). “*Daphnia pulex* Genes 2010 beta 3” annotations, provided by the wFleaBase (http://wfleabase.org) were utilized for the reference-genes-guided steps. Those loci which were located on the filtered out scaffolds were excluded, and the final gene set comprised 41,561 transcripts in total. Intron length boundaries were estimated based on the official gene annotations.

Mapping of the reads was performed independently with TopHat v. 2.0.6 [52] and GSNAP v. 2012-12-20 [53]. In TopHat the following principal options were specified for mapping paired reads: --read-mismatches 7 --read-gap-length 2 --read-edit-dist 7 --mate-inner-dist 0 --mate-std-dev 100 --min-anchor-length 5 --min-intron-length 10 --max-intron-length 50000 --max-insertion-length 3 --max-deletion-length 3 --microexon-search --segment-mismatches 3 --segment-length 18 --no-coverage-search --min-segment-intron 10 --max-segment-intron 50000 --min-coverage-intron 10 --max-coverage-intron 20000 --b2-sensitive --report-secondary-alignments --max-multihits 10. Singleton reads decoupled during read filtering were mapped independently with analogous parameters, except that no new junctions were allowed (--no-novel-juncs), besides junctions, obtained on the first step, as recommended by the developers. Mapping options for GSNAP were as follows: --max-mismatches=0.07 --suboptimal-levels=2 --novelsplicing=1 --localsplicedist=50000 --pairmax-rna=50000 --sam-multiple-primaries with the paired reads and singletons analyzed together.

Transcripts for both mapping approaches were assembled with Cufflinks v. 2.0.2 [54, 55]. The assembly was performed with the following general parameters: --multi-read-correct --upper-quartile-norm --max-intron-length 50000 --min-intron-length 10 --overlap-radius 10 and the bias correction option turned on. Two alternative assemblies were made: *de novo* assembly without predefined annotations and reference-guided assembly (the --GTF option) with cuffmerge and cuffcompare, respectively, used to compare the reconstructed transcripts to the reference.

Mapping-independent *de novo* assembly was done with Trinity r. 2012-10-05 [56]. Input dataset for Trinity was prepared as follows. First, for the paired reads in each pair an attempt was made to merge the mates in one longer fragment with the requirement of at least 15 bases of overlap and allowance of no more than 2 % mismatches, with the fastq-join program from the ea-utils package v. 1.1.2 (http://code.google.com/p/ea-utils). Resulting merged fragments, remaining paired reads and singleton reads were treated as unpaired sequenced and were united together both from the control and treatment with subsequent exclusion of redundant identical sequences by fastx_collapser, a program from the FASTX-Toolkit v. 0.0.13 (http://hannonlab.cshl.edu/fastx_toolkit). Resulting dataset was processed by Trinity with defaults settings, with the exception of butterfly --max_diffs_same_path=5 --min_per_align_same_path=0.95 -SW options and minimal contig length of 60 nucleotides. To obtain expression levels for the Trinity contigs, individual reads were aligned back to them with the aid of Bowtie2 v. 2.0.4 [57] with --end-to-end --sensitive --rdg 7,3 --rfg 7,3 settings separately for the control and the treatment. Resulting mappings were processed by cufflinks with the following parameters: --multi-read-correct --upper-quartile-norm --overlap-radius 10 and the whole contigs specified as predefined annotations, as well as input for bias correction. To define genomic positions of the Trinity contigs, we mapped them to the reference genome with blat (default settings for RNA and minimal identity 93 %). To detect presence and classes of overlaps of the Trinity contigs and the official annotations, cuffcompare from the cufflinks package was used.

Differential expression on the level of genes was assessed with cuffdiff with parameters compatible with those of cufflinks.

### Functional annotations

Two different sources of information on gene functions were used:

- Official annotations as supplied with the “Genes 2010 beta 3” gene set.
- InterProScan [58] motif-based analysis for the whole official gene set was performed with the RunIprScan v. 1.0.0 client (http://michaelrthon.com/runiprscan/) in April 2013.

Accordingly, two sets of gene ontology (GO) assignments were used for GO-term enrichment analysis: from the official gene annotations and from the information provided by InterPro for individual domains and families.

Predictions made with RunIprScan were expanded to all members of the respective families of paralogous genes. These families were defined based on pairwise similarity of the amino-acid sequences of all genes longer than 16 amino-acids with the aid of blat, similar to the approach utilized for detection of potentially indistinguishable cDNA sequences outlined above. A pair of proteins were assigned to the same family if alignment segments with a minimum of 60 % identity covered at least 90 % of the longer protein sequence, which can be considered as a conservative and safe approach (compare with [59]).

Term enrichment analyses for GO-terms, gene families and InterPro domain and family assignments were performed in GOseq v. 1.12.0 [60] for lists of differentially expressed genes obtained with mapping-based approaches only. All genes we weighted by their length and the resulting probability weighting function was used for the over-representation analysis with default settings, independently for up- and down-regulated genes. Only genes with respective annotations (GO classes, gene family membership etc.) were involved in the calculation. Lists of over-represented terms were filtered by controlling false discovery rate at the level of 0.025 within tests.

### Assigning biological function to genes

The multitude of genes without identifiable orthologs in other animal genomes makes correct functional characterization of *Daphnia* genes challenging. In the current work, we overcame this obstacle in two ways. First, we used not only the gene function assignments as reported in the official gene prediction set, but also results from an independent domain-oriented analysis (InterProScan). The second strategy was to expand these functional designations to whole families of paralogous genes defined in a conservative way. This last approach inevitably leads to propagation of false positives, but nevertheless it provides a good starting point for gene function prediction in a situation when no other source of evidence is available. The *D. pulex* genome contains an extraordinarily large number of genes organized in families of paralogs [4]. Given this multiplicity of gene families, even with sensitive mapping-oriented transcriptome assembly, there is always uncertainty about precise locations of the reads coming from genes which are very similar on the mRNA sequence level. This problem becomes even more evident when dealing with *D. pulex* clones different from the one chosen for genome sequencing, as was the case in this study. If not compensated, this can lead to incorrect estimations of differential expression as well as biased results with regard to enrichment analyses. As described in detail below, we decided to select only a single gene from a group if the similarity between their transcripts exceeded a given threshold, 90 % of a pairwise alignment covered by 96 % identical segments.

### Detection of potentially indistinguishable cDNA sequences

Highly similar cDNA sequences were detected with blat [61]: mRNA sequences from the reduced official gene set were used as the query and the target in the same run. blat alignment segments with 96 % identity for individual hits were merged and pairs with overall alignment length at least 90 % were detected as potentially indistinguishable. Individual groups of highly similar genes were created based on the resulting network of pairwise hits. For down-stream analyses only single representatives of the respective groups were utilized.

### Analysis backbone

All of the necessary format conversions and file rearrangements were performed with the aid of SAMtools v. 0.1.18 [62], standard Unix commands, MySQL queries and custom PHP, R and bash scripts. The data and the reference genome were visualized and manipulated on a local installation of the UCSC Genome Browser [63] with the assembly 06.09.2005 of the *Daphnia pulex* genome [4] and associated annotations (raw files were downloaded from the wFleaBase: http://wfleabase.org). MySQL-database for the genome browser was further customized to incorporate the results of our functional annotations and information on the lists of differentially expressed genes (see above) and besides the standard interface was accessed directly with SQL commands and a local web-based search tool. Venn and pie diagrams were constructed with the aid of the VennEuler v. 1.1-0 R package [64] and the SVGGraph library (http://www.goat1000.com/svggraph.php), respectively.

## Results

### RNA-Seq data quality

After the stringent filtering steps, 8.0 and 8.6 million read pairs, as well as 6.7 and 7.5 million singleton reads were retained for the control and treatment libraries, respectively. 58.8–62.5 and 85.1–90.7 % of the reads were successfully mapped to the reference genome by TopHat and GSNAP. On average 19,652–26,008 transcripts encompassed at least one mapped fragment, with the mean number of mapped fragments 441.4–605.6 per transcript. Analysis of the obtained assemblies yielded 288 and 364 differentially expressed (DE) genes for TopHat and GSNAP respectively (see Additional files 1 and 2). The discrepancies between the two mapping methods could not be attributed to the potential differences in read assignment in cases of very similar paralogs as only one such unambiguous case was detected (data not shown). *De novo* reconstruction of full-length transcripts was limited due to moderate read coverage. Therefore, the contigs obtained by the reference-independent assembler Trinity were used only as a supplement to the two mapping-oriented approaches.

### Lists of differentially expressed genes

Reference-independent and reference-guided TopHat and GSNAP assemblies yielded very similar lists of DE genes (data not shown), and all of the DE genes were previously annotated in the reference genome. For further analysis, the lists produced with these assembly strategies were merged producing two united lists for the two mapping programs (referred below as “TopHat” and “GSNAP” lists).

Overall, 435 genes were identified as differentially expressed by at least one approach. The two mapping approaches and the *de novo* assembly yielded similar lists of DE genes. 256 genes shown to be regulated by at least two approaches were considered the strongest candidates for differential expression (see Additional files 1 and 2). From the sets of DE genes we further excluded paralogous genes with nearly identical mRNA sequences, since the actual number of members being differentially expressed cannot be deduced for gene groups with high sequence similarities. A random representative of each group was retained.

**Fig. 2:**
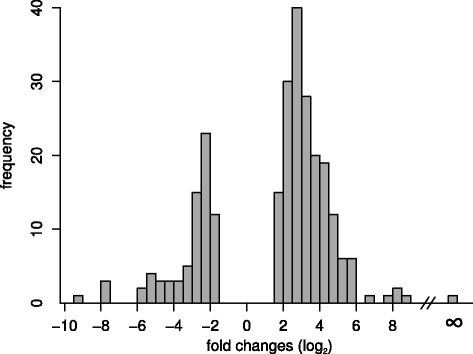
Frequency distribution of fold changes of DE genes. *l* *o* *g*_2_ of the ratio “kairomone treatment”/“control” averaged over three assembly methods: GSNAP, TopHat and Trinity

The list of DE genes identified by at least two approaches is composed of a set of 230 genes: 158 up- and 72 down-regulated in the presence of the kairomone. Distribution of the *log*_2_ fold changes in expression levels for these genes is shown in Fig. 2. Several genes were shown to be regulated in an on/off manner (i.e. showing no expression in either control or treatment) by individual assembly approaches. After averaging over the assemblies this strictly binary regulation was retained for only one of them: *hxAUG26rep1s6g18t1*, a protein without assigned function in the official gene set, for which a mollusc metallothionein family 2 signature was identified by InterProScan (see Additional file 1). Published results generated by qPCR (for predefined candidate genes) and tiled genomic microarrays to examine differentially expressed genes after treating *Daphnia* with *Chaoborus* kairomones [4, 65] were compared with our RNA-Seq dataset. All three lists show a low degree of overlap with the other two sets, but nevertheless the lists compiled from the results of the microarray and RNA-Seq analyses do share 31 genes with concordant patterns of expression, as well as four additional genes which show differential regulation in the opposite direction.

### Functional annotations

To functionally characterize DE genes, we used two independent sources of information: official gene annotations and InterProScan domain predictions.

In many cases even single amino-acid differences precluded domain identification in some otherwise identical proteins. Thus, to increase the power of the enrichment analyses, functional assignments and gene ontologies were interpolated from hits to individual members of paralogs to whole families of paralogous genes. As the gene family assignments provided with the official gene annotations were too broad, we performed independent, more stringent analysis of similarity between protein sequences.

### Over-represented InterPro terms

Table 1 shows significantly over-represented domains and families as identified by InterProScan for the gene sets obtained with the aid of the two mapping methods. Among the up-regulated genes, genes coding for presumed cuticle-associated proteins (with 26–32 of them tagged as “insect cuticle proteins”) are most prevalent. Less abundant are proteins with evidence of lipid transport domains (lipoproteins), vitellogenin domains, and vWF domains, followed by genes coding for globins (together with cruorins) and cyclins.

**Table 1:** Significantly over-represented InterPro domains and families among the differentially expressed genes. In total 46,928 annotations for 18,168 genes were available. The last two columns represent gene counts for significantly over-represented groups as revealed with the aid of the two mapping strategies

Regulation	Type	InterPro ID	Description	Number^a^	GSNAP	TopHat
UP	Domain	IPR001747	Lipid transport protein, N-terminal	22	6	6
UP	Domain	IPR001846	von Willebrand factor, type D domain	24	5	5
UP	Domain	IPR004367	Cyclin, C-terminal domain	11	3	3
UP	Domain	IPR009050	Globin-like	25	4	5
UP	Domain	IPR011030	Vitellinogen, superhelical	18	5	5
UP	Domain	IPR012292	Globin, structural domain	27	4	5
UP	Domain	IPR015255	Vitellinogen, open beta-sheet	12	5	5
UP	Domain	IPR015816	Vitellinogen, beta-sheet N-terminal	21	6	6
UP	Domain	IPR015819	Lipid transport protein, beta-sheet shell	20	6	6
UP	Family	IPR000618	Insect cuticle protein	304	32	26
UP	Family	IPR000971	Globin	23	4	5
UP	Family	IPR002336	Erythrocruorin	13	ns	4
UP	Family	IPR014400	Cyclin A/B/D/E	9	3	3
UP	Family	IPR022727	Pupal cuticle protein C1	5	3	3
DN	Domain	IPR000885	Fibrillar collagen, C-terminal	56	4	4
DN	Domain	IPR001073	Complement C1q protein	172	7	7
DN	Domain	IPR001304	C-type lectin	68	5	4
DN	Domain	IPR002181	Fibrinogen, *α*/*β*/*γ* chain, C-terminal globular domain	50	4	4
DN	Domain	IPR008983	Tumour necrosis factor-like domain	180	7	7
DN	Domain	IPR014716	Fibrinogen, *α*/*β*/*γ* chain, C-terminal globular, subdomain 1	45	4	ns
DN	Domain	IPR016186	C-type lectin-like	83	5	ns
DN	Domain	IPR016187	C-type lectin fold	86	5	ns
DN	Family	IPR002076	GNS1/SUR4 membrane protein	15	3	4

ns — group not significantly over-represented

^a^Total number of genes in the respective category

The list of down-regulated candidates is enriched for genes coding for proteins with lectin-C, CUB, fibrinogen, collagen, TNF-like and complement C1q domains as well as proteins assigned to the GNS1/SUR4 family of unknown molecular function.

### GO-enrichment analysis

Two sets of GO-term assignments were used in the GO-enrichment analysis: one deriving from the official gene annotations and another obtained with domain and family annotations reported by InterProScan. Corresponding lists of over-represented ontologies are shown in Tables 2 and 3. The terms obtained with InterProScan tend to be more precise and some of them have no corresponding categories in the second list, such as “regulation of cell cycle”, as well as the most abundant term pointing to cuticle-associated proteins. Over-representation of yolk proteins (“nutrient reservoir activity”) is explicitly detected as such only with the official annotations. For the down-regulated genes only one term was detected as being significantly over-represented with both annotation sources: carbohydrate binding, with collagen-related terms being additionally represented only in the InterPro-based list.

**Table 2:** Over-represented Gene Ontology terms based on the assignments from InterPro-annotations. In total 40,177 annotations for 14,503 genes were available

Regulation	Ontology^a^	GO ID	Description	Number^b^	GSNAP	TopHat
UP	BP	GO:0006801	Superoxide metabolic process	16	ns	3
UP	BP	GO:0006869	Lipid transport	27	6	6
UP	BP	GO:0015671	Oxygen transport	27	4	5
UP	BP	GO:0051726	Regulation of cell cycle	14	ns	3
UP	CC	GO:0005833	Hemoglobin complex	13	ns	4
UP	MF	GO:0005319	Lipid transporter activity	24	6	6
UP	MF	GO:0019825	Oxygen binding	27	4	5
UP	MF	GO:0042302	Structural constituent of cuticle	304	32	26
DN	CC	GO:0005581	Collagen trimer	58	4	4
DN	MF	GO:0005201	Extracellular matrix structural constituent	59	4	4
DN	MF	GO:0030246	Carbohydrate binding	110	5	ns

ns — group not significantly over-represented

^a^MF: Molecular Function, CC: Cellular Component, BP: Biological Process

^b^Total number of genes in the respective category

**Table 3:** Over-represented Gene Ontology terms based on the GO assignments in the wFleaBase. In total 87,517 annotations for 13,612 genes were available

Regulation	Ontology^a^	GO ID	Description	Number^b^	GSNAP	TopHat
UP	BP	GO:0006810	Transport	1425	18	17
UP	BP	GO:0006950	Response to stress	1028	14	ns
UP	CC	GO:0005576	Extracellular region	1024	15	14
UP	MF	GO:0005198	Structural molecule activity	439	9	9
UP	MF	GO:0005215	Transporter activity	717	13	14
UP	MF	GO:0016209	Antioxidant activity	121	7	8
UP	MF	GO:0019825	Oxygen binding	43	4	5
UP	MF	GO:0045735	Nutrient reservoir activity	8	4	4
DN	MF	GO:0030246	Carbohydrate binding	101	6	5

ns — group not significantly over-represented

^a^MF: Molecular Function, CC: Cellular Component, BP: Biological Process

^b^Total number of genes in the respective category

### Chromatin and cell control proteins

**Fig. 3:**
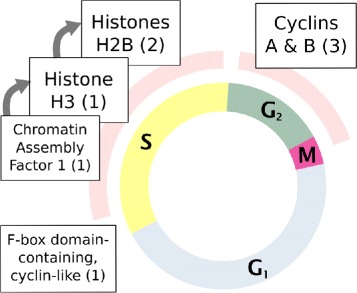
Differentially expressed genes involved in cell-cycle control and chromatin regulation with their relative timing as known for other animals. The internal circle represents cell-cycle. The boxes corresponding to different proteins are arranged according to their role in progressing respective cell-cycle stages. The role of the F-box domain-containing protein can not be predicted with certainty. Numbers in parentheses represent numbers of differentially expressed genes belonging to respective families

Many of the up-regulated genes detected by RNA-Seq code for chromatin-associated or cell-cycle promoting proteins, although not all of the respective functions have been shown to be significantly over-represented (Fig. 3). Among them are nucleosome assembling proteins such as CAF-1, and histones H3 and H2b. Another histone H2b variant, distinct at the mRNA level (84.5 % identity), but similar on the amino-acid level (with the exception of the N-terminus, overall 77.2 % identity) is down-regulated. Cyclins encoded by three up-regulated genes belong to the A (1 gene) and B (2 genes) types.

### Chemoreceptors and hormones

Among the DE genes detected by at least two assembly approaches only one is annotated as a gustatory receptor in the official gene set, *hxAUG25p1s10g327t1* possessing a Scavenger receptor CD36 domain as reported in the wFleaBase and by InterProScan, with a fold change of 6.2 in our experiment. The only protein with identifiable humoral function in the RNA-Seq list of DE genes is an uncharacterized gene *hxAUG26res18g88t1* with insulin-like domains identified by InterProScan. Its expression showed a 7.0 fold increase in the kairomone-treated juveniles (see Additional file 1).

### Over-represented families of paralogs

In the list of genes shown to be differentially expressed by at least two approaches, 27 % of the up-regulated and 22 % of the down-regulated genes belong to families of paralogs. Some of these families are represented by several candidate genes in our list of DE genes and among them a considerable number of families is significantly over-represented (Table 4). The largest family among the up-regulated genes includes genes coding for products similar to a) pupal cuticle proteins, followed by b) Zinc-metalloproteinases, c) vitellogenin, d) a second family of cuticle-associated proteins, e) globin-cruorins, and f) other smaller families (Table 4). Products of gene families with significant down-regulation are characterized as a) C-type lectins, b) proteins of unknown molecular function with similarity to the C1q complement protein, c) proteins involved in elongation of very long chain fatty acids, and d) other less abundant families with nearly (Table 4).

**Table 4:** Over-represented families of paralogous genes based on the wFleaBase annotations. In total 3,978 families encompassed 24,102 genes (genes per family: median — 3, 5–95 % interval — 2–18)

Regulation	Family	Function	Number^a^	GSNAP	TopHat
UP	Omcl36	Pupal cuticle protein	59	11	9
UP	Omcl49	Pupal cuticle protein	47	6	7
UP	Omcl195	Zinc-metalloproteinase	19	9	8
UP	Omcl240	Globin	15	4	5
UP	Omcl335	Vitellogenin/Superoxide dismutase	12	6	6
UP	Omcl886	Cuticle protein	5	3	ns
UP	Omcl2139	Unknown	2	2	2
UP	Omcl3428	Cyclin a	2	2	ns
UP	Omcl3680	Unknown	2	2	2
DN	Omcl23	Neurexin/Complement C1q	82	5	5
DN	Omcl277	Elongation of very long chain fatty acids protein	15	3	4
DN	Omcl329	C-type lectin	12	6	5
DN	Omcl1532	Unknown	3	2	3
DN	Omcl1713	Unknown	3	2	ns
DN	Omcl1963	Unknown	3	3	3
DN	Omcl2758	Unknown	2	2	2
DN	Omcl3591	Unknown	2	2	2

ns — group not significantly over-represented

^a^Total number of genes in the respective family

## Discussion

### Gene expression patterns in the physiological context

Our RNA-Seq results generally agree with the hypothesis proposed in the introduction (see also Fig. 1):

1. The most abundant and significantly over-represented functional group of up-regulated genes is composed of genes coding for cuticle-associated proteins (Tables 1, 2 and 4), which directly echoes morphological observations: i.e., production of neck-teeth (the main defence mechanism of the juvenile *D. pulex*), and changes in cuticle ultrastructure [26].
  In addition, we observed increased transcription of genes involved in chromatin restructuring and the cell cycle (cyclins). This is likely related to the increased proliferative activity recently reported in the region underlying neck-teeth in induced *D. pulex* juveniles [66].
2. A clear hint of resource re-allocation is suggested by the increase in expression of genes involved in lipid transport and metabolism, as well as globins. Six vitellogenin (precursor of the major yolk protein, vitellin [67, 68]) genes belong to this group as well. Production of yolk in daphnids starts as early as late juvenile stages, but the onset of vitellogenin mRNA synthesis takes place even earlier [69–71]. Vitellogenin is synthesized in fat bodies [72]; thus, the presence of residual maternal mRNA in our experiment can be excluded. In this respect, the increased expression of vitellogenin genes seems to point to one of the following factors or their combination: earlier onset of vitellogenesis, increased fecundity, or increased size of progeny. In a physiological study of vitellogenesis in *D. magna*, it was discovered that induction with the *Chaoborus* kairomone has no effect on the onset of yolk production, but decreases its rate [69]. Moreover, in a recent proteomics study of *D. magna* responses to another invertebrate predator, *Triops*, vitellogenin was shown to be among the proteins with decreased production in induced specimens [73]. These observations were based on protein content measurements for a distantly related species producing no neck-teeth, likely explaining theclear contradiction with our results. However,for *D. pulex* it was shown that kairomoneinduction leads to production of bigger offspring [29], which presumably requires a larger poolof yolk.
3. Among functional groups significantly over-represented for the down-regulated genes, we find a large number of genes coding for proteins with domains that play various cellular roles: lectins, and proteins with CUB, fibrinogen and TNF domains. Intriguingly, proteins containing these domains function in immune responses in other invertebrates [74–76]. Although many details of molecular mechanisms of immune responses in Cladocera remain unknown [77, 78], decreased expressionof these proteins may be causally connected tothe observation that inducible defences in*Daphnia* lead to decreased resistance todiseases [79].
4. Potential regulatory genes involved in metabolism of hormones and neurotransmitters or coding for chemoreceptors are nearly absent from our lists of differentially expressed genes. Among the up-regulated genes we found a gustatory receptor, which was designated as such in wFleaBase, but was not reported in an extensive *in silico* study of chemoreceptors in *D. pulex* [46] (see Additional file 1). We speculate that this receptor may be involved in perception of the kairomone, but this requires further experimental evidence.
  No genes with identifiable roles in transcriptional regulation were discovered with our approach, which is likely related to the moderate sequencing coverage of the RNA-Seq.

### Comparison to previous studies, future perspectives

A comparison of the lists of DE genes discovered in *Chaoborus*-induction experiments on juvenile *D. pulex* presented here with the results obtained from tiling microarrays shows that the two lists share a group of 31 genes showing concordant expression patterns. The overall discrepancy is nevertheless noticeable and may be attributed to differences in experimental set-up and/or water conditions and not to the differences in the platform, since comparative analyses generally reveal good correlation between microarray- and sequencing-based techniques [80–82]. Although both experiments were performed on the same *D. pulex* clone, the stage chosen for the microarray experiment was more advanced in comparison to the animals utilized in the current study. This observation signifies the necessity of an experiment involving several developmental stages to sample genes involved in different steps of the predator perception, signal perception and neck-teeth production and maintenance.

It is clear that any results obtained for a single genetic clone should not be over-extrapolated. More experiments on different *D. pulex* clones are necessary to make firm conclusions about the intraspecific variation of the genetic mechanisms acting in the anti-predatory response. Moreover, the existence of neck-teeth producing *Daphnia* species not directly related to *D. pulex* [21] calls for even broader sample of species to investigate the differences in trajectories ultimately leading to similar morphological features.

## Conclusions

This study provides important insights into gene regulatory patterns underlying predator-induced defences, utilizing for the first time unbiased whole-transcriptome RNA-Seq expression data. In particular, our study characterizes different effector genes and gene families underlying morphological and life-history changes which are largely in agreement with expectations based on observed phenotypic changes. Our data represent the largest dataset on the genetic basis of anti-predator defences in *Daphnia* to date and add an important contribution to link a phenotypically plastic response directly with the underlying molecular genetic processes. A deeper understanding of these processes would be achieved with experiments on different genetic clones and at different developmental stages.

## Additional files

Additional file 1
**List of the differentially expressed genes.** List of the 256 differentially expressed genes shown to be regulated in juvenile *D. pulex* as a response to the presence of *Chaoborus* larvae with at least two assembly methods. Gene names, locations and descriptions are according to the official gene annotations as provided by wFleaBase. InterPro term assignments are based on our analysis with the aid of InterProScan. The “+ref” suffixes refer to reference-guided versions of the respective mapping algorithms. Binary Excel File.

Additional file 2
**Venn diagram of DE gene counts as identified by two mapping approaches:** GSNAP **and** TopHat **, and** Trinity ***de novo*** **assembly.**
